# Severe upper gastrointestinal disorders in pembrolizumab‐treated non‐small cell lung cancer patient

**DOI:** 10.1002/rcr2.334

**Published:** 2018-08-01

**Authors:** Tsugitoshi Onuki, Eri Morita, Noritaka Sakamoto, Yoshiaki Nagai, Masafumi Sata, Koichi Hagiwara

**Affiliations:** ^1^ Division of Pulmonary Medicine, Department of Internal Medicine Jichi Medical University Shimotsuke Japan

**Keywords:** Immune checkpoint inhibitor, immune‐related adverse events, non‐small cell lung cancer, pembrolizumab, upper gastrointestinal disorder

## Abstract

Pembrolizumab is an immune checkpoint inhibitor that induces side effects called “immune‐related adverse events” (irAEs). Various types of organs are affected by irAEs, although reports of upper gastrointestinal disorders are rare. Here, we report a case of extensive inflammatory pathologies in the oesophagus, stomach, duodenum, and jejunum after the administration of pembrolizumab for non‐small cell lung cancer.

## Introduction

Pembrolizumab is a monoclonal antibody used in the treatment of multiple neoplasms, including non‐small cell lung cancer (NSCLC). It targets the programmed cell death protein 1 (PD‐1) molecule and is a member of the immune checkpoint inhibitors (ICIs). The side effects of ICIs are referred to as immune‐related adverse events (irAEs), and may affect several organs. IrAEs that occur in the lower gastrointestinal (GI) tract (such as diarrhoea and colitis) have frequently been reported, whereas there are few reports for those in the upper GI tract. Here, we present a case of esophagitis, gastritis, duodenitis, and jejunitis after pembrolizumab therapy for NSCLC.

## Case Report

A 68‐year‐old man was referred to our hospital following for an abnormal shadow in a chest X‐ray. Computed tomography (CT) revealed a nodular lesion in the upper lobe of the left lung measuring 41 mm in longest diameter. A bronchoscopic examination revealed that the lesion was squamous cell carcinoma of the lung. Left upper lobectomy was performed, and the pathological staging was stage IIB (pT2aN1M0). Postoperative adjuvant chemotherapy was performed. After two cycles of cisplatin and vinorelbine, an infiltrative shadow appeared in the right lower lobe. A bronchoscopic examination identified squamous cell carcinoma, which was considered a metastatic lesion. The tumour proportion score identified by the anti‐PD‐L1 antibody test was more than 75%, and pembrolizumab (200 mg once every 3 weeks) was chosen for the treatment. The tumour was in the range of stable disease (SD) after three courses of treatment. No irAEs were observed in the initial six courses.

**Figure 1:**
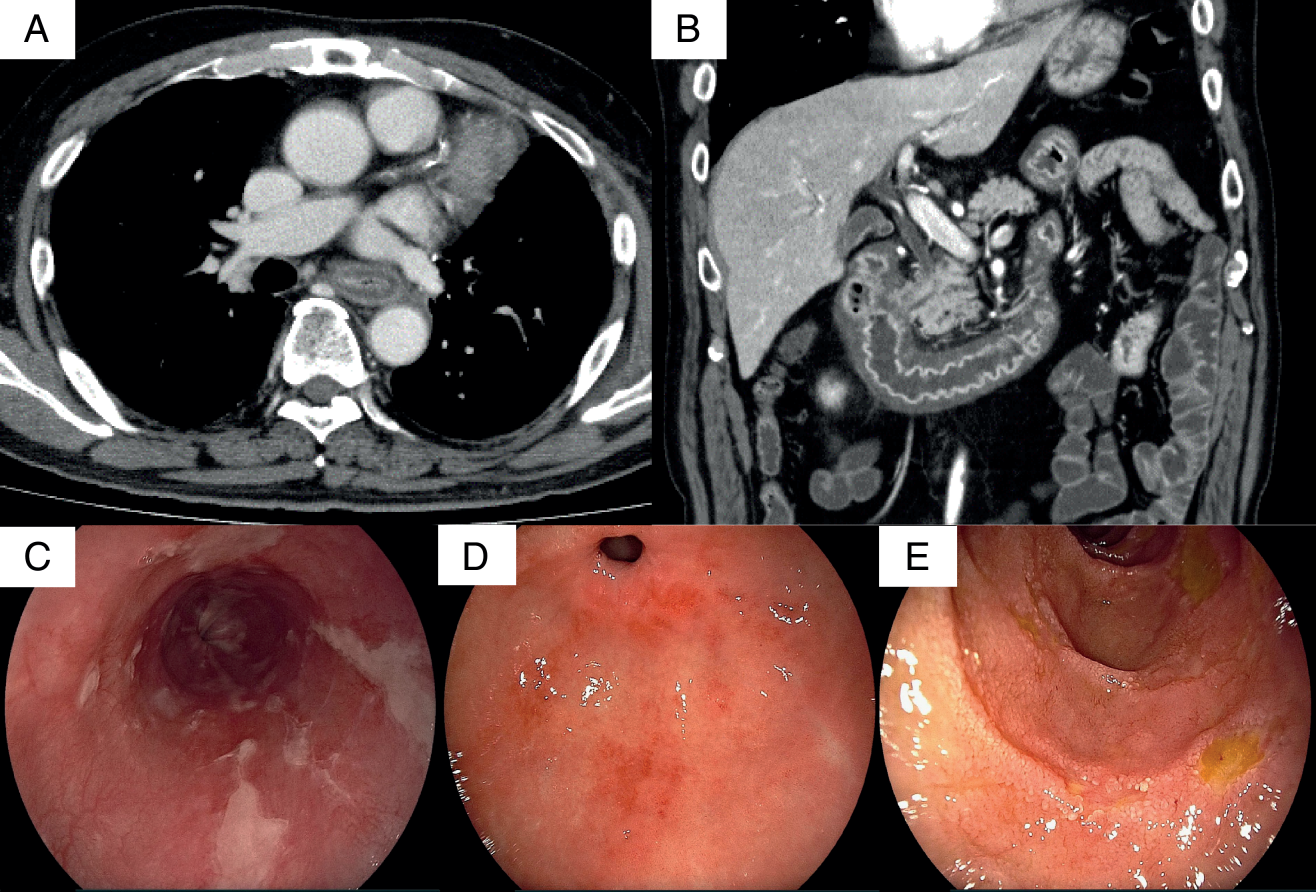
(A and B) A thoraco‐abdominal contrast CT revealed extensive thickening and oedema in the walls of the oesophagus, stomach, duodenum, and jejunum. (C–E) Oesophageal gastroduodenal endoscopy revealed diffuse longitudinal ulcers of the oesophagus (C), which were different from ulcers observed in cases of reflux esophagitis. The stomach demonstrated erosion in the upper part and mottled reddening near the pyloric ring (D). Mucosal atrophy of the stomach was not found. Small ulcers and flares were scattered in the whole duodenum (D).

**Figure 2:**
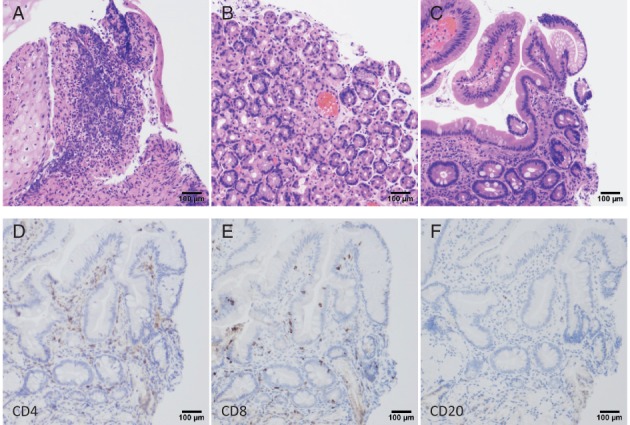
(A–C) Histopathology shows inflammatory cell infiltration based on lymphocytes in the lamina propria. (A) Oesophagus (haematoxylin–eosin, 10×, bar 100 μm). (B) Stomach (haematoxylin–eosin, 10×, bar 100 μm). (C) Duodenum (haematoxylin–eosin, 10×, bar 100 μm). (D–F) Immunostaining shows mainly CD4‐positive (D) and CD8‐positive (E) lymphocytes, with some CD20‐positive cells (F) in the duodenum.

Fever and anorexia occurred on the 14th day of the seventh course, and abdominal pain occurred on the 16th day. On the 21st day, he was hospitalized due to the exacerbation of his abdominal pain. Upon physical examination, he had an elevated body temperature (37.7 °C) and tenderness around the umbilicus. Diarrhoea was not present. Blood tests demonstrated an increase in the white blood cell count (neutrophil dominant) and an elevated C‐reactive protein level. A thoraco‐abdominal contrast CT revealed extensive thickening and oedema in the wall of the upper GI tract (Fig. 1A,B). Oesophageal gastroduodenal endoscopy revealed diffuse longitudinal ulcers of the oesophagus (Fig. 1C). The stomach demonstrated erosion in the upper part and mottled reddening near the pyloric ring (Fig. 1D). Small ulcers and flares were scattered throughout the whole duodenum (Fig. 1E). Biopsies of the oesophagus demonstrated epithelial erosion and subepithelial lymphocytic infiltrates. Gastric and duodenal biopsies demonstrated lymphocyte‐dominant infiltration in the lamina propria, where few atypical epithelia were observed (Fig. 2A–C). Stomach biopsies revealed no histological evidence of eosinophil infiltration, *Helicobacter pylori* infection, or cytomegalovirus infection.

The presence of abdominal pain, the CT, and endoscopic findings suggested an irAE induced by pembrolizumab. Intravenous prednisone (1.0 mg/kg daily) and treatment with an intravenous proton pump inhibitor was initiated. The patient was instructed not to eat until the abdominal pain subsided, and within a few days, his symptoms improved. Specifically, wall thickening and oedema of the upper GI tract were greatly reduced. The steroid dose was gradually reduced and terminated after 5 weeks of administration. Signs and symptoms of irAE did not subsequently recur. Tumour progression was not observed 1 month after we completed steroid therapy.

## Discussion

Here, we presented a case of upper GI disorder after pembrolizumab therapy for NSCLC. There are few reports of upper GI disorders induced by ICIs. Cumulative data from clinical trials of pembrolizumab (*n* = 2266) indicate that the frequency of GI disorder is 26.4% (*n* = 598) and colitis is 1.5% (*n* = 35) (KEYTRUDA® interview form, MSD, December 2017, seventh edition). Esophagitis, duodenitis, or small intestinal inflammation was each observed in one case. There are only two case reports of upper GI disorders by ICIs: Boike et al. reported esophagitis and gastritis after nivolumab therapy for malignant lymphoma 1, and Kobayasi reported gastritis after nivolumab therapy for NSCLC 2.

Symptoms related to lower GI disorders, such as diarrhoea and colitis, appear within 6–9 weeks after the start of drug administration 3. However, these symptoms occurred much later in the studies by Boike and Kobayasi (6 and 4 months, respectively). In our case, symptoms appeared about 6 months after the first administration. Thus, the symptoms of the upper GI disorder may develop later than those of the lower ones. Steroids are the standard for treatment of colitis, whereas the anti‐tumor necrosis factor (TNF)‐α antibody, infliximab, has been used for severe and refractory cases 4. The patient in the present case responded well to steroid therapy.

A study on the clinicopathological findings of 20 patients treated with ICIs 5 reported characteristic findings including a lack of prominent intra‐epithelial lymphocytes and crypt rupture. In a case report of gastritis induced by nivolumab 2, a stomach biopsy specimen showed remarkable lymphocytic cell infiltration and neutrophil infiltration of the mucosa of the fundic gland. Immunostaining identified these lymphocytes as predominantly CD3‐positive T cells, CD4‐positive helper T cells, and CD8‐positive cytotoxicity and suppressor T cells. The mechanisms underlying the pathology of irAEs are unknown, although several mechanisms have been proposed. For example, cell and tissue injury may be due to self‐reactive CD8‐positive T cells. Alternatively, cells and tissues may be damaged by plasma antibody‐mediated autoantibody production from CD4‐positive T cells. The composition of lymphocytes observed in our case is similar to a previous report, where CD4‐positive and CD8‐positive lymphocytes predominated, and some CD20‐positive cells were observed (Fig. 2D–F). Immunostaining may help the diagnosis in the upper GI disorder induced by ICIs.

In conclusion, we report a case of upper GI disorder induced by pembrolizumab, including image findings, endoscopic findings, pathological findings, and course of treatment.

### Disclosure Statement

Appropriate written informed consent was obtained for publication of this case report and accompanying images.
